# Comparative Performance of Anyplex II HPV28 and Cobas 4800 Human Papillomavirus (HPV) Assays for High-Risk HPV Detection in Self-collected Anal Samples

**DOI:** 10.1093/ofid/ofad540

**Published:** 2023-10-31

**Authors:** Gauthier Delvallez, Sokleaph Cheng, Stéphane Marot, Gervillien Arnold Malonga, Théophile Cocherie, Steve Wignall, Vincent Calvez, Sophat Phal, Kem Vichet, Anne-Geneviève Marcelin, Aude Jary

**Affiliations:** Medical Biology Laboratory, Institut Pasteur du Cambodge, Phnom Penh, Cambodia; Medical Biology Laboratory, Institut Pasteur du Cambodge, Phnom Penh, Cambodia; Sorbonne Université, Inserm, Institut Pierre Louis d’Epidémiologie et de Santé Publique, Assistance Publique–Hôpitaux de Paris, Service de Virologie, Hôpital Pitié-Salpêtrière, Department of Virology, Paris, France; Sorbonne Université, Inserm, Institut Pierre Louis d’Epidémiologie et de Santé Publique, Assistance Publique–Hôpitaux de Paris, Service de Virologie, Hôpital Pitié-Salpêtrière, Department of Virology, Paris, France; Sorbonne Université, Inserm, Institut Pierre Louis d’Epidémiologie et de Santé Publique, Assistance Publique–Hôpitaux de Paris, Service de Virologie, Hôpital Pitié-Salpêtrière, Department of Virology, Paris, France; Family Health International, Phnom Penh, Cambodia; Sorbonne Université, Inserm, Institut Pierre Louis d’Epidémiologie et de Santé Publique, Assistance Publique–Hôpitaux de Paris, Service de Virologie, Hôpital Pitié-Salpêtrière, Department of Virology, Paris, France; Family Health International, Phnom Penh, Cambodia; Men's Health Cambodia, Phnom Penh, Cambodia; Sorbonne Université, Inserm, Institut Pierre Louis d’Epidémiologie et de Santé Publique, Assistance Publique–Hôpitaux de Paris, Service de Virologie, Hôpital Pitié-Salpêtrière, Department of Virology, Paris, France; Sorbonne Université, Inserm, Institut Pierre Louis d’Epidémiologie et de Santé Publique, Assistance Publique–Hôpitaux de Paris, Service de Virologie, Hôpital Pitié-Salpêtrière, Department of Virology, Paris, France

**Keywords:** anal lesions, Anyplex II HPV-28, Cobas 4800, HPV testing, self-sampling

## Abstract

We compared 2 human papillomavirus (HPV) assays to detect the 14 high-risk HPV (hrHPV) genotypes in self-collected anal samples. We found a good agreement and similar performance to detect HPV-16, HPV-18, and the 12 other hrHPV genotypes. The global performance to detect the 14 hrHPV genotypes was not significantly different between the 2 assays.

Around 30 000 new cases of anal squamous cell carcinoma (ASCC) are diagnosed each year worldwide [1]. Key populations, such as men who have sex with men (MSM) and people with human immunodeficiency virus (HIV), have an increased risk of developing ASCC. For instance, HIV-infected MSM have the highest incidence rate of ASCC with 85 new cases per 100 000 person-years [2]. Human papillomavirus (HPV) is involved in roughly 90% of ASCCs, of which 90% are HPV-16 positive. ASCC is preceded by precancerous lesions—that is, anal intraepithelial neoplasia 1, 2, and 3 (AIN1, AIN2, and AIN3, respectively), with AIN1 being identified as low-grade anal lesions and AIN2 and AIN3 as high-grade anal lesions (HGAIN). However for now, high-risk HPV testing (hrHPV) is not recommended for the screening of anal precancerous and cancer lesions, whereas it has been clinically validated with multiple hrHPV tests in cervical cancer screening [3, 4]. Although anal HPV-16 genotyping is not yet a widely accepted molecular marker in anal cancer screening, it is by far the most carcinogenetic type in the anus [5]. Indeed, HPV-16 prevalence increases across the anal lesion from normal to precancerous and anal cancer [6], and in a recent prospective study, HGAIN HPV-16–positive cases are less likely to clear compared to those with other hrHPV genotypes [7].

In this study, we aim to compare the agreement and performance of the Cobas 4800 HPV test (Roche) and the Anyplex II HPV28 Detection assay (Seegene Inc) to detect hrHPV in self-sampling anal swabs from asymptomatic MSM and transgender women (TGW) in Phnom Penh, Cambodia, especially for HPV-16 detection, which was the most prevalent HPV genotype found in this population [8].

## MATERIALS AND METHODS

### Study Population

A cross-sectional retrospective study was conducted between 1 October 2021 and 30 November 2021 in different clinics in Phnom Penh, Cambodia, and under the scope of activities of Men's Health Cambodia, a nongovernmental and nonprofit organization working with men, MSM, and TGW in Cambodia. During consultation, a questionnaire was used to collect patient data, and anal collection (FLOQSwabs, Copan) was self-performed using anal swabs discharged in viral transport media (VTM). They were then stored at −80°C at the Institut Pasteur du Cambodge (IPC) until further analysis. A comprehensive explanation of eligibility criteria and procedures has been published previously [8].

### HPV Testing and Methods Comparison

First analysis was performed at IPC on 400 μL of sample with the Cobas 4800 HPV test (Roche), which allows the detection of 14 hrHPV types by polymerase chain reaction (PCR) and nucleic acid hybridization. This test specifically identifies HPV-16, HPV-18, and 12 other hrHPV types in the same channel including HPV types 31, 33, 35, 39, 45, 51, 52, 56, 58, 59, 66, and 68. Then, 1 mL of frozen VTM was sent to the Virology Department of the Pitié-Salpêtrière Hospital, Paris, France. DNA extraction (from 200 μL of sample to 100 μL) was performed with the E-mag device (bioMérieux), and both samples and extracted DNA were stored at −80°C. Then, HPV testing was performed with the Anyplex II HPV28 Detection assay (Seegene Inc), which detects and differentiates 28 distinct HPV types including the 14 hrHPV types detected by the Cobas 4800 HPV test and 14 other HPV types (not analyzed in this work). Both HPV tests also detect a human gene as internal control to assess the quality of the sampling and the efficiency of the PCR amplification. If internal control was not amplified, samples were referred as invalid.

Statistical analysis was performed with the package *rstatix* from R software. McNemar and kappa indices were calculated to compare the proportion of positive samples and the degree of agreement between the 2 methods to detect the 14 hrHPV types, respectively. A 2-tailed *P* value of <.05 was considered significant.

## RESULTS

Overall, 162 participants were included with a median (IQR) age of 28.1 (24.4–31.8) years, of whom 56% were MSM and 8% were HIV infected. None of them received any HPV vaccine.

One hundred sixty-two anal samples were tested with both the Roche and Seegene methods, and HPV testing failed for 59 (36%) and 41 (25%) samples, respectively. The prevalence of HPV-16, HPV-18, and other hrHPV types was not significantly different between the 2 methods: 20% versus 25% (*P* = .52), 16% versus 17% (*P* = .86), and 50% versus 59% (*P* = .18), respectively (Table 1).

**Table 1.** Prevalence of Human Papillomavirus (HPV) Types 16 and 18 and Other High-Risk HPV Types Found With the 2 Assays

HPV Type	Seegene (n = 121)^a^	Roche (n = 103)^b^	*P* Value^c^
HPV-16, No. (%)			
Negative	91 (75)	82 (80)	.52
Positive	30 (25)	21 (20)	
HPV-18, No. (%)			
Negative	101 (83)	87 (84)	.86
Positive	20 (17)	16 (16)	
Other hrHPV types^d^, No. (%)			
Negative	50 (41)	52 (50)	.18
Positive	71 (59)	51 (50)	

Abbreviations: HPV, human papillomavirus; hrHPV, high-risk human papillomavirus.

^a^Forty-one samples being referred as invalid with Seegene HPV test.

^b^Fifty-nine samples being referred as invalid with Roche HPV test.

^c^Fisher exact test.

^d^HPV types 31, 33, 35, 39, 45, 51, 52, 56, 58, 59, 66, and 68.

Further statistical analyses for method comparison were performed on 97 samples, 65 samples being referred as invalid in 1 or both of the 2 methods. The concordance of the results for detection of HPV-16, HPV-18, and other hrHPV type was 97%, 100%, and 88%, respectively (Table 2). Fifteen samples were discordant: (*i*) 3 for HPV-16 positivity, of which 2 HPV-16 were missed by Roche and 1 missed by Seegene; and (*ii*) 12 for other hrHPV positivity, of which 8 hrHPV types were missed by Roche and 4 by Seegene (Supplementary Table 1). HPV-52 was the most common hrHPV type missed by Roche (6/10 cases), followed by HPV-16 and HPV-56 (2/10 cases each). For most of the hrHPV types missed by Roche, they were detected tardily by Seegene, after 40 cycles of amplification.

**Table 2.** Agreement and Performance of the 2 Methods to Detect Human Papillomavirus (HPV) Types 16 and 18 and Other High-Risk Types

HPV Type	No. (%) (n = 97^a^)	Cohen Kappa Test (95% CIs)	McNemar Test
HPV-16, No. (%)			
Concordant	94 (97)	0.91 (0.81–1.00)	1.00
Discordant	3 (3)	…	…
HPV-18, No. (%)			
Concordant	97 (100)	1.00 (1.00–1.00)	NA
Discordant	0 (0)	…	…
Other hrHPV types^b^, No. (%)			
Concordant	85 (88)	0.75 (.62–.88)	0.39
Discordant	12 (12)	…	…
All hrHPV types, No. (%)			
Concordant	90 (93)	0.85 (.74–.96)	0.44
Discordant	7 (7)	…	…

Cohen Kappa test and McNemar test were performed to compare the agreement and the performance of the 2 methods to detect HPV-16, HPV-18, the other hrHPV types, and the 14 hrHPV types as a whole.

Abbreviations: HPV, human papillomavirus; hrHPV, high-risk human papillomavirus; NA, not Applicable ; 95% CIs, 95% confidence intervals.

^a^Sixty-five samples being referred as invalid in 1 or both HPV tests.

^b^HPV types 31, 33, 35, 39, 45, 51, 52, 56, 58, 59, 66, and 68.

The agreement (Cohen kappa) for detection of HPV-16, HPV-18, and other hrHPV types was 0.91 (95% confidence intervals) (.81–1.00), 1.00 (1.00–1.00), and 0.75 (.62–.88), respectively. The performance to detect HPV-16, HPV-18, and other hrHPV positivity were not significantly different between the 2 tests (McNemar test, *P* > .05) (Table 2 and Supplementary Table 2). In total, the global performance to detect any of the 14 hrHPV types was not significantly different (McNemar test, *P* = .44).

## DISCUSSION

To our knowledge, this is the first study conducted in self-collected anal samples that compared the Cobas 4800 HPV test (Roche) and the Anyplex II HPV28 Detection (Seegene) tests for hrHPV detection.

First, comparing the 2 methods on 97 samples revealed a good agreement between them and comparable worldwide ability to identify any of the 14 hrHPV genotypes currently recommended for cervical cancer screening. This result was also consistent for HPV-16 alone, HPV-18 alone, and the other hrHPV genotypes detected with the 2 methods. However, compared to the Seegene approach, the Cobas test missed more anal hrHPV infections. In term of hrHPV prevalence in normal cervical tissue, a difference was also identified between the Cobas method and GP5^+^/GP6^+^ PCR testing followed by genotyping with Luminex [4]. However, equal frequency was showed in samples with abnormal cytology. In our work, we dealt with anal samples and we did not assess the association with cytology results; thus, these 2 factors may have an impact on the difference between the 2 procedures.

The prevalence of HPV-16 was the highest in our high-risk population, as expected, and the 2 methods showed high agreement and performance to detect the most oncogenic genotype in the anal site. This is of importance as HPV-16 testing could be one of the biomarkers used as anal cancer screening in key populations in the near future. For instance in France, new guidelines propose to use HPV-16 testing in anal cytology specimen collected by physicians as an anal cancer screening marker in MSM living with HIV [9]. If positive, a reflex cytology is performed on the same sample and patient is referred to a proctologist.

Similarly to self-collected vaginal swabs for cervical cancer screening [10], self-collected anal samples could be of interest to reach patients outside the healthcare system [11]. In our work, 41 (25%) and 59 (36%) samples failed to be amplified by the Anyplex II HPV28 Detection assay and Cobas 4800 HPV test, respectively. Low cellularity due to inappropriate sampling and misunderstanding of the participants may explain this high rate of invalid results. If implemented in this population, self-collection instructions should be improved.

In conclusion, both the Anyplex II HPV28 Detection assay and the Cobas 4800 HPV test are suitable to detect the 14 hrHPV genotypes, in particular HPV-16, in self-collected anal samples.

## Supplementary Material

ofad540_Supplementary_Data

## Supplementary Data

Supplementary materials are available at *Open Forum Infectious Diseases* online. Consisting of data provided by the authors to benefit the reader, the posted materials are not copyedited and are the sole responsibility of the authors, so questions or comments should be addressed to the corresponding author.

## Notes

***Author contributions*.** Conception and design of the study: A. J., S. C., and G. D. Acquisition of the clinical data: S. W., S. P., and K. V. Execution of the experiments: G. A. M. Analysis of the data: A. J., S. M., and G. D. Drafting of significant portion of the manuscript or figures: A. J., V. C., A. G. M., and G. D. All authors read, corrected, and approved the final version of the manuscript.

***Patient consent*.** Each patient's written consent was collected, and permission for the study was obtained from the National Ethics Committee for Health Research in Cambodia (approval letter number 189NECHR).

***Financial support*.** This study was funded by the Institut Pasteur du Cambodge and the Agence Nationale de recherche sur le SIDA et les hépatites virales–Maladies infectieuses émergentes.
