# Supplementary material for: Comparative Performance of Anyplex II HPV28 and Cobas 4800 Human Papillomavirus (HPV) Assays for High-Risk HPV Detection in Self-collected Anal Samples
Source: Open Forum Infect Dis. 2023 Oct 31;10(11):ofad540. doi: 10.1093/ofid/ofad540 (PMC10656044; doi:10.1093/ofid/ofad540)
Supplement: ofad540_Supplementary_Data [file ofad540_supplementary_data.docx]

**Supplementary Table 1** Description of the 15 samples with discordant results between Roche and Seegene HPV assays

| **Participant ID** | **Discordance** | **HPV-types** | **Amplification***  **(Seegene)** | **Missed by** |
| --- | --- | --- | --- | --- |
| HPV-236 | HPV16 | HPV16 | NA | Seegene |
| HPV-020 | HPV16 | HPV16 | (+) | Roche |
| HPV-174 | HPV16 | HPV16 | (+) | Roche |
| HPV-171 | others hrHPV | HPV51 and HPV52 | 51 (+), 52 (+) | Roche |
| HPV-137 | others hrHPV | HPV52 | (+) | Roche |
| HPV-186 | others hrHPV | HPV52 | (++) | Roche |
| HPV-231 | others hrHPV | HPV52 | (+) | Roche |
| HPV-157 | others hrHPV | HPV52 and HPV56 | 52 (++), 56 (++) | Roche |
| HPV-170 | others hrHPV | HPV52 and HPV56 | 52 (++), 56 (+) | Roche |
| HPV-108 | others hrHPV | HPV59 | (+) | Roche |
| HPV-102 | others hrHPV | HPV66 | (+) | Roche |
| HPV-009 | others hrHPV | unknown | NA | Seegene |
| HPV-017 | others hrHPV | unknown | NA | Seegene |
| HPV-172 | others hrHPV | unknown | NA | Seegene |
| HPV-191 | others hrHPV | unknown | NA | Seegene |

NA: not applicable

Others hrHPV: 31, 33, 35, 39, 45, 51, 52, 56, 58, 59, 66 and 68

*+ : detection between 40 and 50 Ct, ++: detection between 30 and 40 Ct, +++ : detection before 30 Ct

HPV: human papillomavirus; hrHPV: high-risk human papillomavirus

**Supplementary Table 2:** Agreement of the two methods to detect HPV16, HPV18 and others hrHPV types.

hrHPV others: 31, 33, 35, 39, 45, 51, 52, 56, 58, 59, 66 and 68

HPV: human papillomavirus; hrHPV: high-risk human papillomavirus

|  |  | **SEEGENE** | |  |
| --- | --- | --- | --- | --- |
| **ROCHE** | **HPV16** | Positive test | Negative test | Total |
|  | Positive test | 20 | 1 | 21 |
|  | Negative test | 2 | 74 | 76 |
|  | Total | 22 | 75 | 97 |
|  | **HPV18** | Positive test | Negative test | Total |
|  | Positive test | 16 | 0 | 16 |
|  | Negative test | 0 | 81 | 81 |
|  | Total | 16 | 81 | 97 |
|  | **others hrHPV** | Positive test | Negative test | Total |
|  | Positive test | 45 | 4 | 49 |
|  | Negative test | 8 | 40 | 48 |
|  | Total | 53 | 44 | 97 |
|  | **All hrHPV** | Positive test | Negative test | Total |
|  | Positive test | 55 | 2 | 57 |
|  | Negative test | 5 | 35 | 40 |
|  | Total | 60 | 37 | 97 |
